# The Latest Developments in Immunomodulation of Mesenchymal Stem Cells in the Treatment of Intrauterine Adhesions, Both Allogeneic and Autologous

**DOI:** 10.3389/fimmu.2021.785717

**Published:** 2021-11-15

**Authors:** Jia-ming Chen, Qiao-yi Huang, Yun-xia Zhao, Wei-hong Chen, Shu Lin, Qi-yang Shi

**Affiliations:** ^1^ Department of Gynaecology and Obstetrics, The Second Affiliated Hospital of Fujian Medical University, Quanzhou, China; ^2^ Department of Gynaecology and Obstetrics, Shenzhen Hospital of University of Hong Kong, Shenzhen, China; ^3^ Centre of Neurological and Metabolic Research, The Second Affiliated Hospital of Fujian Medical University, Quanzhou, China; ^4^ Diabetes and Metabolism Division, Garvan Institute of Medical Research, Sydney, NSW, Australia

**Keywords:** immunoregulation, allogeneic MSCs, autologous MSC, intrauterine adhesion, rejection reaction

## Abstract

Intrauterine adhesion (IUA) is an endometrial fibrosis disease caused by repeated operations of the uterus and is a common cause of female infertility. In recent years, treatment using mesenchymal stem cells (MSCs) has been proposed by many researchers and is now widely used in clinics because of the low immunogenicity of MSCs. It is believed that allogeneic MSCs can be used to treat IUA because MSCs express only low levels of MHC class I molecules and no MHC class II or co-stimulatory molecules. However, many scholars still believe that the use of allogeneic MSCs to treat IUA may lead to immune rejection. Compared with allogeneic MSCs, autologous MSCs are safer, more ethical, and can better adapt to the body. Here, we review recently published articles on the immunomodulation of allogeneic and autologous MSCs in IUA therapy, with the aim of proving that the use of autologous MSCs can reduce the possibility of immune rejection in the treatment of IUAs.

## Introduction

Intrauterine adhesion (IUA), also known as Asherman syndrome, is condition involving endometrial fibrosis caused by damage to the basal layer of the uterus, leading to partial or full adhesion of the uterine cavity (1, 2). IUA is usually accompanied by decreased menstrual flow, and in severe cases, secondary amenorrhea. Moreover, IUA affects embryo implantation and development as a result of reduction or even complete disappearance of intrauterine volume, leading to female infertility and recurrent miscarriage (3). However, normal endometrium is not scarred during repair. Under the influence of uterine manipulation and inflammatory cytokines, the endometrium participates in hypoxia, reduces new blood vessels, and controls the expression of adhesion-related cytokines (4).

At present, hysteroscopic endometrial adhesion decomposition combined with various methods, such as insertion of an intrauterine balloon (5), administration of hyaluronic acid gel (6) and polyethylene oxide-sodium carboxymethylcellulose gel (2), etc., can be used to treat IUA. However, the common adhesion release can only improve the problem of uterine cavity stenosis and cannot completely reverse endometrial fibrosis. Therefore, researchers have proposed the use of ​​ stem cells to treat IUA because of their regenerative ability (7).

Recently, Cao et al. (8) reported that the use of umbilical cord MSCs in allogeneic cell therapy could improve the treatment of recurrent uterine adhesions, and subsequently conducted a phase I clinical trial. Since umbilical cord MSCs have low immunogenicity and low tumorigenicity, they have great advantages in the application of IUA. On the one hand, clinical trials showed that endometrial thickening and IUA score after treatment were lower than those before treatment. On the other hand, DNA short tandem repeat (STR) analysis showed the regenerated endometrium only containing patient DNA, so that umbilical cord MSCs exhibit good safety. Mare (9) also confirmed that allogeneic MSCs can regulate the protein pattern and increase the proliferation of glandular epithelial cells, thereby exerting an anti-scarring effect. Although the immunogenicity of MSCs is lower than that of other stem cells, immune rejection of allogeneic MSCs can still occur. Ankrum et al. (10) pointed out that antibodies and immune rejection may be produced during the treatment of allogeneic donor MSCs. In other words, MSCs may not have immune privileges.

Currently, the mechanism by which allogeneic mesenchymal stem cells induce immune rejection remains unclear. Most researchers believe that the human leukocyte antigen (HLA) of allogeneic MSCs does not match the receptor type (11). Others have suggested that immune rejection caused by allogeneic MSCs is also related to immune cells (12), immunoactive substances (3, 13) and immune organs (14). Currently, there is no good method to control immune rejection. A prior study suggested (10) that the immune persistence of MSCs could be improved, and immune tolerance could be enhanced. More importantly, others have suggested (11) that knockout of beta-2 microglobulin could be performed to enhance repair. The most direct approach is the use of autologous MSCs. Kim et al. believe (15) that the combination of MSC spheres and autologous composite sheets could be used to avoid immune rejection and improve the effectiveness of stem cell therapy. This article reviews the comparison between allogeneic and autologous MSCs. In addition, we will summarize the mechanism by which allogeneic mesenchymal stem cell therapy induces immunoregulation. Furthermore, we will discuss the therapeutic prospects of autologous MSCs.

## Intrauterine Adhesion

**Figure 1:**
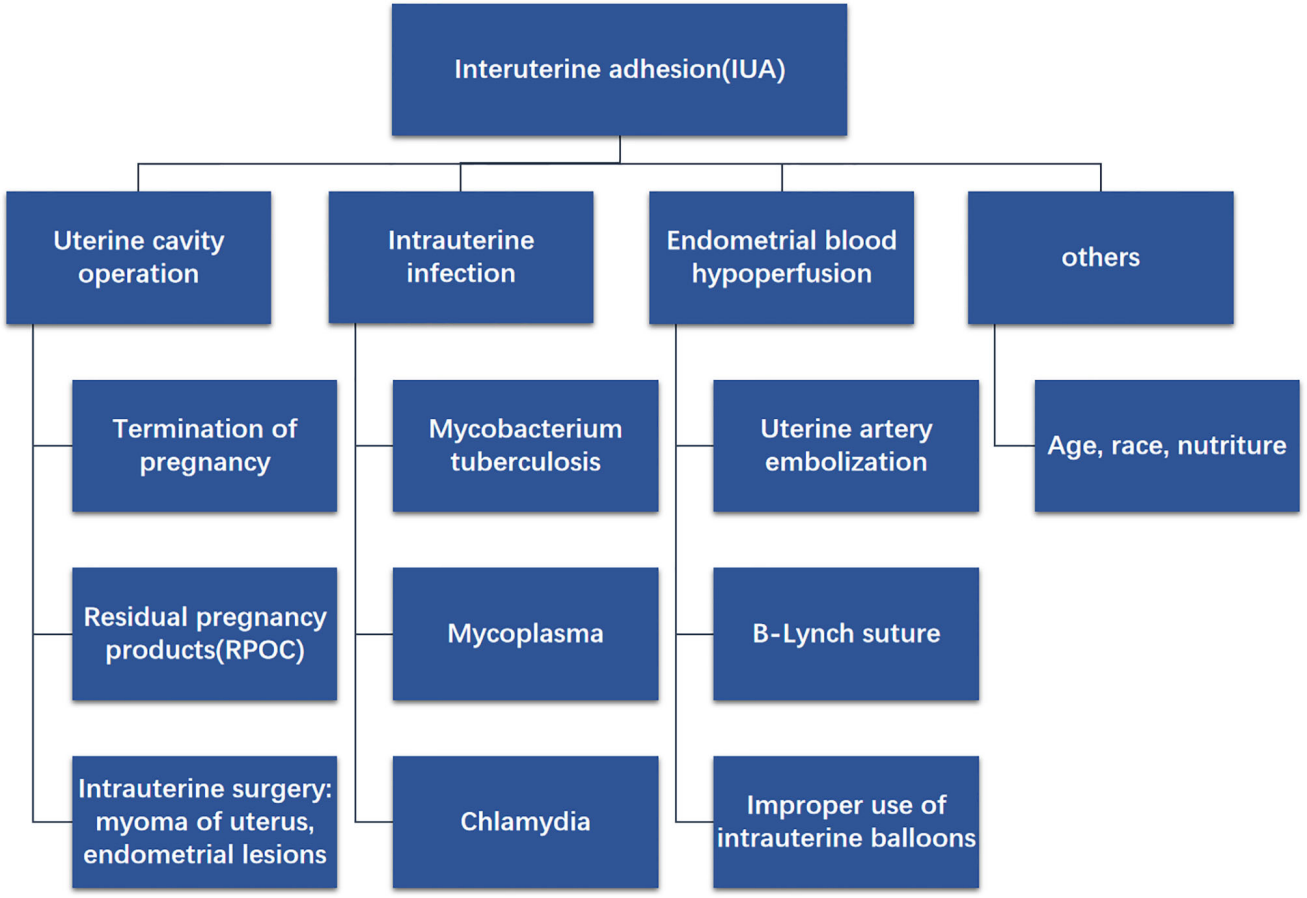
Diagram shows the possible causes of IUA, for example uterine cavity operation, intrauterine infection, endometrial blood hypoperfusion and others.

IUA often progresses as a consequence of uterine cavity operation. Deans et al. conducted (16) a retrospective analysis of 1856 patients with intrauterine adhesions and found that 67% of curettage was induced or spontaneous, while 22% was due to postpartum bleeding. Christina et al. (17) considered that antagonistic effects occur due to increased prolactin levels and decreased estrogen levels in the postpartum period; therefore, the endometrium is more prone to atrophy. Additionally, IUA is associated with inflammatory cytokines. The experimental results of Mo et al. (18) demonstrated that preoperative inflammation in IUA patients was significantly higher than that in non-IUA patients. At present, the only confirmed infectious factor that causes IUA is genital tuberculosis (4). Research has shown (19) that *Mycobacterium tuberculosis* infection of the uterine cavity can lead to focal ulceration, necrosis, or bleeding of the endometrial tissue, while destruction of the endometrium can cause partial or complete IUA. Another important reason is the insufficient endometrial blood perfusion (20). For example, uterine artery embolism, B-Lynch suture, hysteroscopic myomectomy, etc. (21). Due to the long-term insufficient blood supply to the endometrium, it is difficult to regenerate, which increases the possibility of IUA (**Figure 1**).

**Figure 2:**
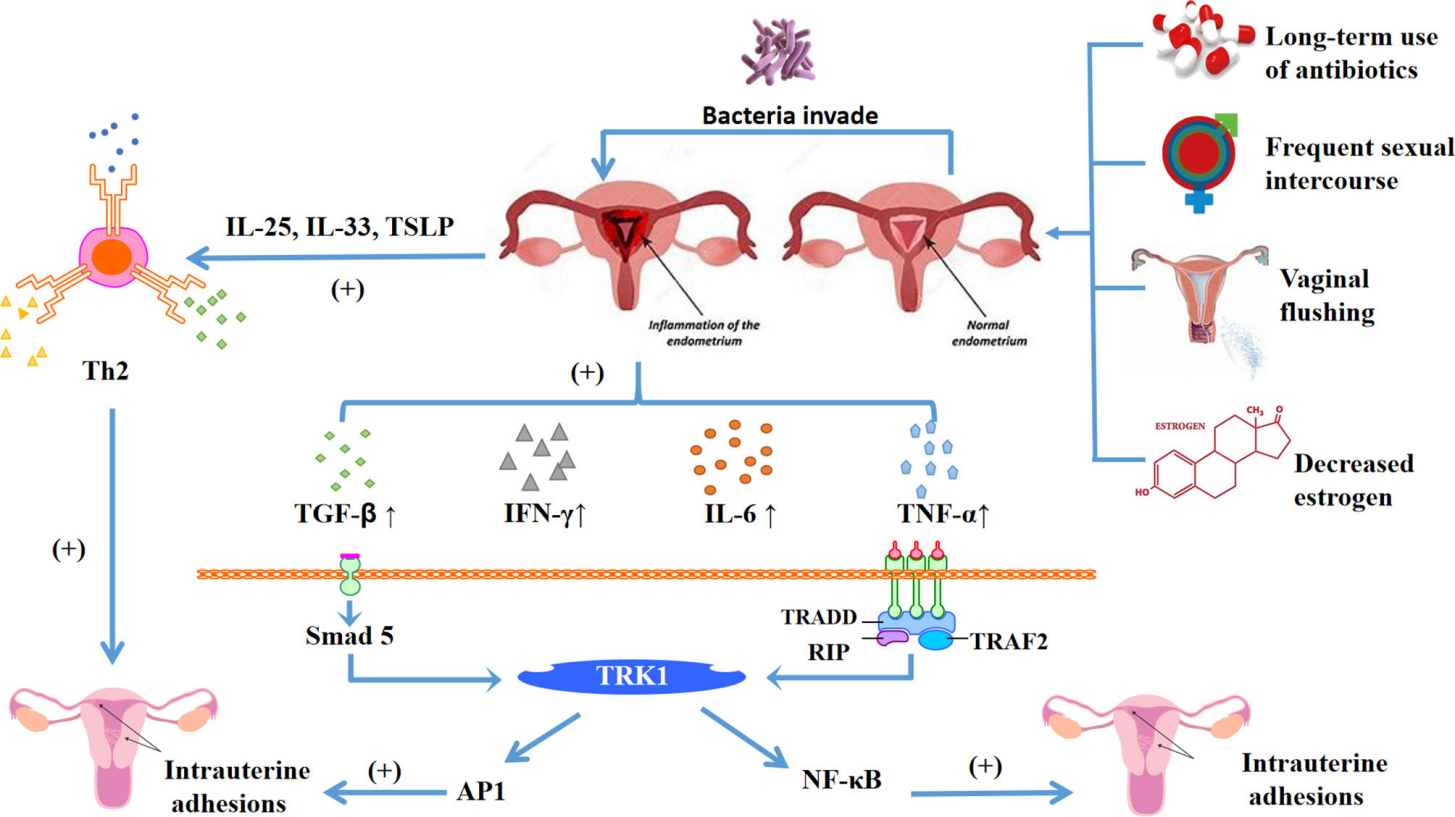
Diagram shows the main mechanism of intrauterine adhesions, the damaged endometrium is affected by long-term antibiotic treatment, frequent sexual intercourse, vaginal flushing, decreased estrogen and other factors, which lead to the homeostasis of the internal environment changing. Broken rings in the microenvironment make bacteria easier to invade, and the damaged endometrium has an inflammatory response. On the one hand, it can release inflammatory cytokines IL-25, IL-33, TSLP, activate helper Th2 cells, and promote endometrial fibrosis. On the other hand, it can also release inflammatory cytokines TGF-β, IFN-γ, IL-6, TNF-α, etc. Among them, TGF-β can be through TGF-β-Smad5 pathway, and TNF-α and NF-κB jointly promote endometrial fibrosis, thereby forming intrauterine adhesions.

At present, the immune mechanism of intrauterine adhesions is not clear, Zhao et al. (22) believe that it is related to the imbalance of vaginal flora and microecology. The causes of microecological imbalance may be related to the long-term use of antibiotics, frequent sexual intercourse, vaginal flushing, and decreased estrogen. These factors greatly increase the incidence of uterine inflammation. When the endometrium is normal, it can resist the invasion of these inflammatory cytokines. However, once the endometrium is damaged due to uterine operation, the surface barrier is destroyed. On the one hand, bacteria invade the endometrium, causing local inflammatory reactions, and the pro-inflammatory cytokines IL-6 and IFN-γ increase. Negative feedback reduced the activity of matrix metalloproteinase (MMP) and promoted the generation of endometrial fibrosis. On the other hand, damaged epithelial cells (23) release IL-25, IL-33, TSLP, other cells can directly or indirectly promote Th2 immune response to promote fibrosis. What’s more, NF-κB (24) transcription cytokine promotes the expression of intrauterine adhesion inflammatory cytokines and plays a central role in inflammatory diseases. It also closely intersects with the pathogenic cytokines of intrauterine adhesion such as TGF-β, TNF-α, IL-1 and IL-18. Wang et al. (25) found that the expression of NF-κB in endometrium from patients with endometrial adhesion was significantly higher than that in normal endometrium (**Figure 2**).

Currently, the commonly used clinical treatment method is transcervical resection of adhesions (TCRA). To improve the efficacy of the IUA, an intrauterine contraceptive device and intrauterine balloon can be inserted first (26). Intrauterine devices and balloons can expand the narrow uterine cavity to improve the long-term prognosis of patients. In addition, we can also increase biological barriers, such as new cross-linked hyaluronan gel (27), auto-cross-linked hyaluronan gel (28), and estrogen gel (29). However, the prognosis of IUA is still not ideal. Some researchers have postulated (21) that the degree of recurrence of IUA is related to the degree of adhesion. In addition, Hanstede et al. (30) compared the degree of IUA and the recurrence rate of patients with hysteroscopic adhesiolysis. The results indicated that the recurrence rate of first-degree IUA was 3.8% (n=24), that of second-degree IUA was 33.1% (n=211), and that of third-degree IUA was 35.4% (n=226).

Furthermore, the release of adhesions also needs to promote the regeneration and repair of endometrial fibrosis. Due to the regenerative ability of stem cells, researchers are considering the use of stem cells for treatment. Studies have shown (31) that collagen scaffolds loaded with human umbilical cord mesenchymal stem cells can promote endometrial structural reconstruction and functional recovery (**Table 1**).

**Table 1:** This table clearly summarizes some treatment methods for IUA, including hyaluronic acid, mesenchymal stem cells and their combined application.

P value	Results		Measurement index	Model	Treatment	Source
	Control group	Experimental group				
P<0.01, n=6	560 ± 20μm	710 ± 60μm	Endometrial thickness	SD Rat	Collagen scaffolds loaded with human umbilical cord MSCs	Liaobing Xin et al. (31)
	70 ± 3%	43 ± 3%	Area of collagen staining			
	2.1 ± 0.4%	13 ± 2%	ERα			
P<0.01, n=6	3.6320 ± 1.0060	4.9662 ± 1.4935	Uterine glands	Monkey	Human umbilical cord–derived MSCs and autocrosslinked hyaluronic acid gel	Lingjuan Wang et al. (32)
	14.2131 ± 13.7193%	5.5955 ± 3.6572%	Fibrosis area			
	1.0776 ± 0.6650mm	4.2667 ± 0.5558mm	Endometrial thickness			
p=0.0008, n=625	↓ SMD -0.68 95% CI -1.08 ˜ -0.28		IUA scores after miscarriage	Human	Hyaluronic acid gel	Zheng Fei et al. (33)
p=0.0001, n=625	↓ RR 0.44 95% CI 0.29 ˜ 0.67		The incidence of postoperative intrauterine adhesions after miscarriage			
P=0.0012, n=137	33/137(24.1) IUA	13/137(9.5%) IUA	Postoperative efficacy	Human	New Crosslinked Hyaluronan Gel	Xueying Li et al. (27)
	RR 0.3939 95% CI 0.2107–0.7153					
P=0.0006, n=137	1.07 ± 2.06	0.33 ± 0.106	Adhesion scores			
P<0.05, n=20	4.18 ± 0.91mm	9.12 ± 1.78mm	Endometrium thickness	Human	Transdermal estrogen gel and oral aspirin combination	Yugang Chi et al. (29)
	3 ± 0.81	5 ± 1.71	Menstrual flow (days)			
	40.76 ± 8.92	69.72 ± 8.01	Menstrual volume (ml)			
P<0.05, control n=6; experiment n=8	d30:560 ± 50 μm	d30:800 ± 200 μm	Endometrial thickness	SD Rat	A scaffold laden with MSCs-derived exosomes	Liaobing Xin et al. (34)
	d60: 480 ± 80 μm	d60:700 ± 200 μm				
	d30:1 ± 2	d30: 30 ± 10	Uterine glands			
	d60:4 ± 5	d60: 20 ± 20	ERα			
	60 ± 30 cells/mm^2^	400 ± 100 cells/mm^2^				
	6.55%	30.76%	Pregnancy rates			
	400 ± 100 cells/mm^2^	800 ± 100 cells/mm^2^	immunostaining			

The therapeutic effect was evaluated by endometrial thickness, number of uterine glands, endometrial fibrosis area, and postoperative menstrual volume.

The arrows mean that Hyaluronic acid gel reduce the intrauterine adhesion scores after miscarriage (SMD -0.68, 95% CI -1.08∼-0.28; p=0.0008) and the incidence of postoperative intrauterine adhesions after miscarriage (RR 0.44, 95% CI 0.29∼0.67; p = 0.0001).

## Mesenchymal Stem Cells

### Background of Mesenchymal Stem Cells

Mesenchymal stem cells (MSCs) are pluripotent stem cells which can attach to the wall where they grow and replicate (35). The sources of MSCs are relatively abundant; for example, they can be extracted from the bone marrow, umbilical cord, adipose tissue, peripheral blood, and so on (36). MSCs have become a research hotspot for their diverse functions, which include cell differentiation and proliferation, regulation of inflammatory processes, control of oxidative stress, and angiogenesis (37).

### Types of Mesenchymal Stem Cells

Currently, there are several classifications of MSCs; donor MSCs can be classified as either allogeneic or autologous. Allogeneic MSCs are derived from other donors for implantation into the recipient patient. Because they are extracted from a foreign body, the number of allogeneic MSCs can be quite large. However, since allogeneic MSCs are equivalent to foreign matter, they are not ethically recognized in humans (38). In contrast, autologous MSCs are cells that are extracted from the patient’s own tissues, induced and differentiated, and then re-implanted for treatment. Therefore, they adapt better to the patient’s body, and are less likely to suffer from immune rejection. In the past decade (39), genetic modification of autologous hematopoietic stem cells has been used for the treatment of single-gene diseases. Moreover, they are more in line with people’s ethical requirements and are recognized by more people. At present, autologous MSCs have been used to treat several diseases, including multiple sclerosis (40), post-burn scar treatment (41), refractory rectovaginal Crohn’s fistulas (42), and patients with advanced tumors (43), among others (**Table 2**).

**Table 2:** This table mainly compares the differences between autologous MSCs and allogeneic MSCs from five aspects: source, application, ethics, advantages and disadvantages.

Type	Allogeneic MSCs	Autologous MSCs
Source	allogeneic tissues	Autologous tissue: including dental (44),human umbilical cord (45), bone marrow (46), adipose tissue (47)
Application	Hip Osteoarthritis (48) Acute Respiratory Distress Dyndrome (49) Aortic Aneurysms (50) Traumatic Brain Injury (TBI) (51)	Multiple Sclerosis (40) Post-burn Scars Treatment (41) Refractory Rectovaginal Crohn’s Fistulas (42) Patients With Advanced Tumors (43)
Ethic	do not conform to human ethics	conform to human ethics
Advantages	Low acquisition cost and large quantity	High proliferation activation rate High security Low immunogenicity No ethical issue
Shortcomings	Immune rejection High pollution rate Genetic risk Easy tumorigenic	Small quantity *In vitro* amplification takes longtime

The results show that there is little difference between autologous MSCs and allogeneic MSCs in source and application, but autologous MSCs are more ethical, and compared with allogeneic MSCs, they can reduce immune rejection.

In addition, MSCs can be classified by source, as either dental pulp MSCs (44), human umbilical cord MSCs (45), bone marrow MSCs (46), and adipose tissue-derived MSCs (47). In addition, if they are classified by developmental potency, they can be divided into totipotent stem cells (TSCs), pluripotent stem cells, and unipotent stem cells.

### Characteristics of MSCs

MSCs have many notable characteristics. First, MSCs cannot differentiate and self-renew (52). Hsiao et al. (53) proposed that treatment with undifferentiated MSCs can improve insulin resistance in diabetic rats, rebalance inflammation, and improve blood sugar levels. Regarding the differentiation ability of MSCs, researchers have suggested that MSCs can differentiate into adipocytes, osteoblasts, and chondrocytes. In addition, detection of MSC surface antigens indicated that the expression of markers such as CD166, CD44, CD29, CD73, CD90, and CD105 increased significantly with the increase of transmission algebra (20). Moreover, MSCs also express CD11, CD14, CD45, and CD34. However, as the transmission algebra increases, these markers are reduced or lost. Therefore, MSCs can be detected using the above-mentioned markers.

## Mechanism and Application of MSCs in the Treatment of IUA

### MSC Immunization

Current research indicates that application of bone marrow MSCs can promote endometrial regeneration by suppressing the innate and adaptive immune systems (54). On the one hand, in the innate immune system, MSCs can inhibit the activation, proliferation and cytotoxicity of natural killer cells (NK cells) (55). Conversely, in adaptive immunity, MSCs (56) can inhibit the proliferation of B and T cells, and further prevent the proliferation and differentiation of T cells into pro-inflammatory TH1 and TH17 helper T cells. In addition, MSCs can promote the differentiation of T cells into tolerant Treg cells (57), and can regulate the action of monocytes, dendritic cells, and NK cells by secreting chemokines (58), such as IGF-1, TGF-β, bFGF, HGF, IL-6, SDF-1, M-CSF, VEGF, PIGF, and MCP-1 (55).

Toll-like receptors (TLR) are also involved in the immunotherapy of MSCs. When faced with a danger signal, TLRs recognize danger signals and are activated to trigger a cellular response, mobilizing innate and adaptive immune cells (59). The results showed that MSCs combined with TLR4 promoted the secretion of pro-inflammatory cytokines. In contrast, if combined with TLR3, MSCs promote the secretion of anti-inflammatory cytokines (60). When microorganisms are infected, exogenous danger signals such as endotoxins or lipopolysaccharides (LPS) disappear. Meanwhile, abnormal or damaged cells overflow into the circulatory system, resulting in the expression of heat shock proteins, an endogenous danger signal. Get rid of the “danger signal” to activate the sentinel innate immune cells (for example, dendritic cells) TLR. Various immune cells are recruited to the site of endometrial damage (61).

Moreover, researchers have found that the concentration of macrophages in the endometrium of patients with IUA decreased, which may be related to a decrease in CSF-1 (62). Otherwise, another study indicated (63) that transplantation of Human amniotic epithelial cells (HAECs) can be performed through autophagy-induced recovery of damaged endometrium. VEGF expression in the menstrual and proliferative phases can promote the reconstruction of endometrial tissue. HAECs can increase the expression of VEGF, promote angiogenesis, and promote the recovery of endometrial fibrosis.

### Differentiation Therapy of MSCs

Mesenchymal stem cells have the capability to differentiate After MSCs reach the tissue, they secrete stem cell cytokines such as SCF and M-CSF to reactivate the differentiation potential of the endogenous stem cells of the damaged tissue, thus promoting the generation of new tissue cells to replace damaged cells, resulting in tissue recovery; this is termed stem cell differentiation therapy (64). At present, MSC differentiation has been applied in various fields. A set of clinical treatment data of Ashman syndrome showed (65) that patients receiving autologous CD9 ^+^, CD44 ^+^, CD90 ^+^ bone marrow MSC transplantation experienced improved endometrial vascularization and an increase in endometrial thickness. One such patient subsequently underwent *in vitro* fertilization and embryo transfer, and successfully carried a fetus to term.

Differentiation therapy of MSCs can also be applied to other aspects, such as the treatment of Parkinson’s disease, in which EDSCs are promoted to differentiate into dopamine-producing cells (66). Studies have shown (67) that CD90, CD146, and PDGFR-β in HEDSCs can differentiate into neuron-like cells. Moreover, they can also differentiate into cholinergic neuron-like cells (68), oligodendrocyte-like cells (69), insulin-producing cells (70), cardiomyocyte-like cells (71), megakaryocyte-like cells (72), and urothelial cells (73).

### Stem Cell Paracrine Therapy

MSCs coordinate tissue recovery by releasing soluble paracrine cytokines (74). Treatment of IUA through the paracrine pathway is currently the most popular research method as IUA is partially affected by inflammatory cytokines. One of the subgroups of inflammatory cytokines is IL-1ra in the acute inflammatory paracrine effect of MSCs. IL-1ra inhibits cytokine stimulation of the helper T lymphocyte system, as well as the production of the inflammatory cytokines TNF-α in macrophages in an IL-1ra-dependent manner (74).

Furthermore, because exosomes can make mesenchymal stem cells specialize in the treatment of certain diseases, they are now an active field. Exosomes are small, single-membrane secretory organelles with a diameter of 30–200 nm (75). Recent studies have shown (76) that the function, targeting, and mechanism-driven accumulation of specific cellular components in exosomes indicate that they play a role in regulating intercellular communication.

At present, exosomes are also used in the IUA. Yao et al. (77) demonstrated that exosomes from MSCs can reverse the epithelial to mesenchymal transition (EMT) through action of the TGF-β1/Smad pathway and promote the repair of damaged endometrium in 64 female rabbits. Liao et al. (78) also summarized the manner by which different exosomes could be used to treat female reproductive disorders, and concluded that MSCs can improve female fertility in *in vitro* and *in vivo* models. Therapeutic mechanisms include angiogenesis, immune regulation, anti-fibrosis, and anti-oxidative stress. In addition, after subcutaneous injection of a mixture of matrix gel and MSCs in mice, the number of hemoglobin or CD31^+^ cells increased, indicating that MSCs can promote the formation of functional capillaries (79). Furthermore, Zhao et al. (80) demonstrated that ADSC-ex promotes endometrial regeneration and fertility recovery. Moreover, Tao et al. (81) found through experiments that Mir-29a in bone marrow MSC exosomes can inhibit fibrosis in the process of endometrial adhesion repair.

### Homing Characteristics of Stem Cells

The homing function of stem cells functions to recruit stem cells by disrupting the tissue-secreted chemokine system (82). It is well known that chemokine SDF1 and its special receptor CXCR4 play a key role in the homing of BMSCs (83). Moreover, studies have shown that ERα promotes the proliferation and migration of BMSCs through SDF1/CXCR4 (84). Furthermore, the CXCL12/CXCR4 protein ligand is a chemokine receptor complex that can transmit stem cells to the uterus (85), and thus promote endometrial repair. In addition, G-CSF has been shown to mobilize hematopoietic stem cells from the bone marrow into the blood, thereby reducing migration to the endometrium (86). G-CSF can also enhance the expression of cytokeratin, vimentin, integrin, and leukemia inhibitory cytokine (LIF) and regulate endometrial function (7).

## Immunoregulation of Allogeneic MSCs

It is well known that the immunoregulation and regeneration characteristics of MSCs are the reason why they are used to treat many diseases. Because bone marrow MSCs are considered to have low immunogenicity, their implantation does not trigger histocompatibility disorders or predict possible immune rejection (87). The low immunity of MSCs is due to the lack of expression of major histocompatibility complex II (MHCII) and the low expression of costimulatory molecules such as MHCI, CD80, and CD86 (88).

Studies have shown that under certain conditions, bone marrow MSCs can secrete proinflammatory cytokines and act as antigen-presenting cells to promote immune responses (89). Bone marrow MSCs can effectively reduce phagocytosis and antigen presentation of monocytes/macrophages, and promote the expression of immunosuppressive molecules such as interleukin IL-10 and programmed cell death 1 ligand 1 in these cells (90). In addition, they can effectively inhibit the maturation of dendritic cells and their ability to produce pro-inflammatory cytokines, in addition to stimulating strong T-cell responses (90). Furthermore, MSCs can inhibit the generation and pro-inflammatory properties of CD4 ^+^ T helper cells 1 (Th_1_) and Th_17_ cells, and promotes the proliferation and inhibition of regulatory T cells. Bone marrow MSCs can also damage the expansion, cytokine secretion, and cytotoxic activity of pro-inflammatory CD8 ^+^ T cells (90).

### Allogeneic MSCs and Monocytes/Macrophages

Immunomodulatory macrophages can be categorized as either pro-inflammatory M_1_ and anti-inflammatory M_2_ macrophages (91). Some scholars have pointed out that MSCs can effectively promote the polarization of macrophages into the M_2_ type. This process is mediated by a variety of soluble molecules, including PGE2, IDO, HGF, IL-1RA, TSG6, TGF-β, etc. (90). In addition, MSCs induce M2 polarization, the anti-inflammatory cytokines IL-10, arginase-1, and TGF-β increase, and the pro-inflammatory cytokines TNF-α, IL-12, and IL-1 decrease, inhibit T cell response, and induce the production of regulatory T cells. Finally, this leads to further immunosuppression, which is conducive to the treatment of allogeneic MSCs (92).

**Figure 3:**
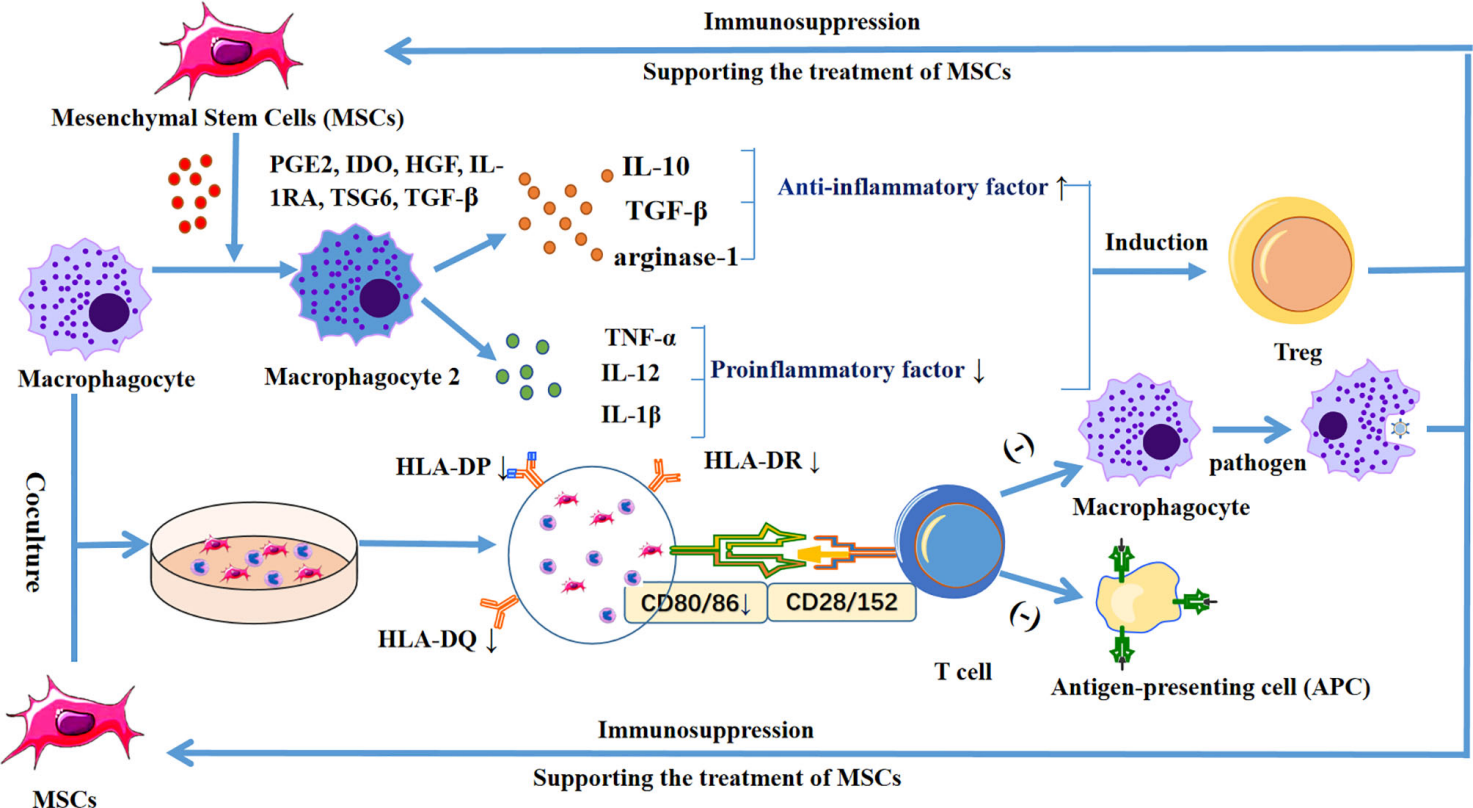
Diagram shows the release of PGE_2_, IDO, HGF, IL-1RA, TSG6, and TGF-β by MSCs, which stimulates macrophages to differentiate into M2 macrophages, increases anti-inflammatory cytokines IL-10, TGF-β, and arginase-1, and decreases the pro-inflammatory cytokines TNF-α, IL-12, and IL-1β, inhibiting the T cell response, and inducing regulatory T cells. In addition, when MSCs were co-cultured with macrophages, the expression levels of HLA-DR/DP/DQ and CD86 were decreased, thereby reducing the phagocytic ability and antigen presentation ability of macrophages, leading to further immunosuppression and supporting the therapeutic effect of MSCs.

Furthermore, when human umbilical cord-derived MSCs (UC-MSCs) are co-cultured with monocytes and macrophages, the expression of HLA-DR/DP/DQ and CD86 is reduced, and the phagocytosis and antigen presentation ability of monocytes and macrophages is decreased (93). This indicates that, compared with ordinary stem cells, UC-MSCs can reduce the chance immune rejection caused by allogeneic transplantation. In addition, US-MSCs can induce CD14+, CD16+, and CD206+ in mice after being phagocytosed by monocytes, leading to an increase in the anti-inflammatory cytokines IL-10 and PD-L1 (94) (**Figure 3**).

### Allogeneic MSCs and Dendritic Cells

Dendritic cells (DCs) are antigen-presenting cells that can induce T cells to produce an immune response. MSCs can inhibit the maturation of dendritic cells by regulating the expression of HLA-DR, CD40, OX40L, CD80, and CD83 (95), and upregulating the expression of PD-L1 (96), thereby reducing its ability to activate T cells. In addition, when DCs are co-cultured with MSCs, the pro-inflammatory cytokines secreted by CD1c+ DCs are reduced, and the anti-inflammatory cytokine IL-10 secreted by plasmacytoid DCs is increased (97). Moreover, studies have shown that exosomes released by adipose-derived mesenchymal stem cells can inhibit IL-6 and increase the expression of the anti-inflammatory cytokines IL-10 and TGF-β (98). Furthermore, treatment of mouse dendritic cells with MSC-derived exosomes did not stimulate T cell proliferation after LPS activation (98)

### Allogeneic MSCs and T Cells

In the T cell family, CD4 ^+^ helper T cells and CD8 ^+^ cytotoxic T cells play important roles in the immune regulation of MSCs. On the one hand, CD4 ^+^ T cells can bind to CD40 and CD40 ligands on their cell surface, thereby enhancing the ability of dendritic cells to induce CTL. On the other hand, CD40 can maintain CTL activity by triggering the secretion of IL-2 (99). However, MSCs inhibit the proliferation of these two tesla cells through paracrine and cell contact, thereby reducing the release of the pro-inflammatory cytokines TNF-α and IFN-γ, and reducing the immune rejection between allogeneic MSC receptors (100).

**Figure 4:**
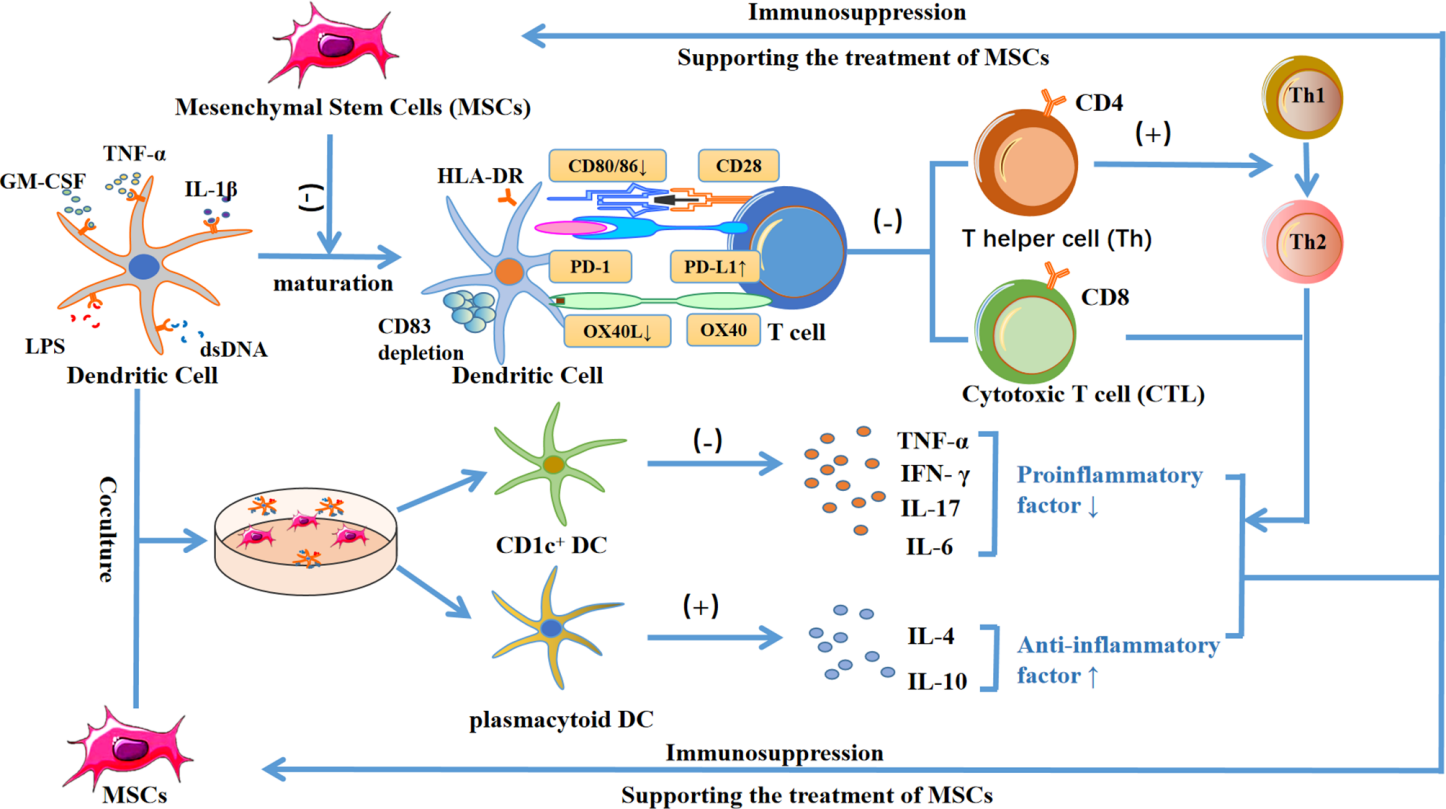
Diagram showing the mechanism by which MSCs inhibit the maturation of dendritic cells, reduce the ability to activate T cells, and decrease HLA-DR, CD40, OX40L, CD80, CD83, CD86 expression, while increasing PD-L1 expression. This process causes the inhibition of CD4^+^ and CD8^+^ T cells by paracrine or cell contact. On the one hand, inhibiting CD4^+^ helper T cells can inhibit the binding of CD40 ligands with CD40 on the surface of dendritic cells, thereby reducing the transformation to CD8^+^ T cells. In addition, inhibiting CD4^+^ helper T cells can reduce the release of IL-2 and formation of CD8 ^+^ T cells, and ultimately reduce the release of pro-inflammatory cytokines TNF-α and IFN-γ. On the other hand, MSCs can induce the differentiation of regulatory T cells and trigger dendritic cells to promote the transformation of Th1 to Th2, resulting in a decrease of the pro-inflammatory cytokines TNF-α, IFN-γ, IL-17 and IL-6, and an increase in the levels of the anti-inflammatory cytokines IL-4 and IL-10, which contributes to the treatment of MSCs. In addition, when dendritic cells were co-cultured with MSCs, the secretion of pro-inflammatory cytokines by CD1c^+^ DC decreased, and the secretion of anti-inflammatory cytokine IL-10 by plasmacytoid DC increased, which further led to immunosuppression and supported the treatment of MSCs.

In addition, exosomes extracted from mouse MSCs can inhibit T cell proliferation by upregulating cyclin-dependent kinase inhibitor 1 B and downregulating cyclin-dependent kinase 2 (101). Bone marrow mesenchymal stem cells can transform Th1 to Th2 mediated by dendritic cells and induce Tregs (56), reduce pro-inflammatory cytokines IFN-α, IL-17, and IL-6, and increase the anti-inflammatory cytokines IL-4 and IL-10 (102). Moreover, exosomes extracted from umbilical cord-derived mesenchymal stem cells can inhibit CD8 + T cells and Th1 cells, reduce the secretion of pro-inflammatory cytokines IFN-γ and TNF-α, induce Tregs, and promote the secretion of the anti-inflammatory cytokines IL-10 (103) (**Figure 4**).

## Immune Rejection Reaction Between Allogeneic and Autologous Mesenchymal Stem Cells

Chronic rejection is the most serious major adverse reaction after allogeneic transplantation, and is also the main obstacle affecting patient survival (104). Due to the presence of alloreactive T cells in the allograft, the allograft recipient usually experiences an acute graft-versus-host response (GVHR) (105–108). Possible mechanisms of this response include host tissue injury, increased secretion of pro-inflammatory cytokines (TNF-α, IFN-γ, IL-1, IL-2, IL-12), and activation of dendritic cells (DCs), macrophages, NK cells, and cytotoxic T cells (109). Because of the low immunogenicity of human mesenchymal stem cells (HMSCs), some scholars have proposed (97) that BMSCs do not express the costimulatory molecules B7-1, B7-2, CD40, and CD40 ligands, and thus may not activate allogeneic reactive T cells.

However, in recent years, some scholars have proposed that although the immunogenicity of mesenchymal stem cells is low, thus fact alone does not explain why mesenchymal stem cells have “immune privileges” (10). Allogeneic MSCs transplanted into the body can still trigger immune rejection; however, at present, it is unclear whether this rejection has a major impact on the treatment effect. This article provides a detailed description of the above-mentioned factors that may affect host immune rejection.

### Host Tissue Injury

In the treatment of osteoarthritis, Djouad et al. (110) initially proposed that although allogeneic mouse MSCs can be transplanted into mice with strong immunity to form bone, lymphocyte infiltration can be seen around newly formed tissues. Fischer et al. (111) also found more severe graft-versus-host disease (GVHD) in mice receiving allogeneic hematopoietic stem cell transplantation than in wild-type mice in clinical models. The main reason explaining this is that during allogeneic hematopoietic stem cell transplantation, damaged or dead epithelial cells release endogenous risk signals and are perceived by pattern recognition receptors on antigen-presenting cells, triggering the release of proinflammatory cytokines and T cells from major donor sources, which attack and destroy host cells and tissues, causing graft-versus-host disease (112).

In addition, studies have shown that the survival rate of allogeneic mesenchymal stem cells is limited when transplanted into the body. Liesz et al. proposed that even in uninjured adult brains, transplanted MSCs can be rejected within 14 days (113). This indicates that after allogeneic MSCs are transplanted into the body, the survival rate is still not guaranteed and some cannot coexist with the host. There are also reports that pigs (114), rats (115) and baboons (116) have dissolved allogeneic antibodies after inoculating allogeneic mesenchymal stem cells, which further confirmed the possibility of immune rejection between allogeneic and autologous MSCs.

### Increased Secretion of Pro-Inflammatory Cytokines and Activation of Immunocytes

Barnhoorn et al. (117) showed that the titer of anti-donor immunoglobulin G increased significantly 7 days after the subcutaneous injection of IFN-γ-activated allogeneic mesenchymal stem cells in pigs. Furthermore, in the use of MSCs to treat traumatic brain injury, it has been proposed (54) that allogeneic mesenchymal stem cells can be actively rejected by the host immune response, mainly due to the cytotoxic CD8+ T cell-mediated response, which limits the therapeutic effect. Melissa et al. (51) released Fas ligand (FasL) through agarose gel to induce the apoptosis of cytotoxic CD8^+^ T cells, thereby reducing immune rejection induced by allogeneic mesenchymal stem cells during transplantation.

In addition, CD4^+^ Tm cells have been shown to induce allograft rejection by activating cytotoxic CD8^+^ T cells and helping B cells produce allogeneic antibodies (118, 119). Moreover, some scholars have found (120) that activated CD4 + T cells secrete IL-2 and IFN-γ, thereby damaging the structure of the extracellular matrix, precipitating extracellular collagen, promoting the proliferation of fibroblasts, and ultimately leading to immune rejection. It has also been suggested (121) that CD4^+^ helper T cells and monocytes can be recruited into the neointima and secrete IL-1, IL-6, and TNF-α, which enables smooth muscle cells to migrate and proliferate in the elastic layer of the endometrium, ultimately leading to graft-versus-host reaction (GVHR).

## Current Treatment of Allogeneic MSCs Immune Rejection

At present, immune rejection of allogeneic mesenchymal stem cells still occurs. Some scholars have proposed that treatment can be refined by improving the persistence of mesenchymal stem cells and the immune tolerance of mesenchymal stem cells (10).

### Improve the Persistence of MSCs

Recent advances have shown that solid organ transplantation can induce mixed hematopoietic chimerism in allogeneic MSC transplantation through donor hematopoietic stem cell transplantation (122). Moreover, the immunization isolation membrane device is used to temporarily prevent rejection by wrapping differentiated allogeneic cells in collagen or gelatin gels. Zanotti et al. have shown that subcutaneous injection of bone marrow MSCs wrapped in alginate can significantly improve their survival in GVHD mice (123).

In addition, in order to overcome the immune rejection caused by transplantation of allogeneic mesenchymal stem cells into the body and prolong the survival time of allogeneic mesenchymal stem cells, it is also necessary to ensure their persistence through host and MSC modification. For example, mesenchymal stem cells can be pre-treated or loaded with biological agents to increase the expression of surface receptors or the production of immunosuppressive cytokines (124).

### Improving the Immune Tolerance of MSCs

In addition to improving immune tolerance, we can improve the efficacy of allogeneic MSCs by extending their survival time *in vivo*. On the one hand, the immune tolerance of allogeneic MSCs can be improved by treatment with exosomes or secreting nutritional and immunoregulatory cytokines to mediate hit-and-run mechanisms (125). However, it has also been proposed that the immune system can be reprogrammed through apoptotic bodies, thereby prolonging the duration of allogeneic MSCs after injection.

### Induction Therapy

Induction therapy can be divided into T cell consumption and T cell non-consumption strategies. The former involves anti-thymocyte globulin (ATG), anti-CD3 antibody (OKT3), and alemtuzumab, which reduce the release of the proinflammatory cytokines TNF-α and IFN-γ by consuming T cells. While anti-IL-2 antibodies such as daclizumab, brazillicoxib, and anti-CD20 antibodies such as rituximab and anti-cytotoxin are considered non-depleting agents for T cells (126). IL-2, IL-6, and IL-1 are autophagy-related cytokines (63), and anti-IL-2 antibodies can reduce immune rejection by reducing autophagy induction.

## New Direction of IUA Treatment: Autologous MSC

Autologous MSCs are safer to use because they use their own stem cells to avoid being attacked by B and T cells. In the treatment of IUA, in order not to affect subsequent fertility, the use of autologous MSCs is more in line with ethical requirements. Therefore, an increasing number of people are currently advocating the use of autologous MSCs for the treatment of female IUA. Such treatment reduces immune rejection, thereby reducing the recipient’s attack on the graft, while the female reproductive system avoids the controversy of potential genetic changes. In the treatment of intrauterine adhesions, this would also guarantee female fertility in later periods.

In 2011, a case study reported the implantation of autologous BMSCs into the uterine cavity in patients with severe IUA at 8 weeks of pregnancy (127). In 2014, a study found that the thickness of the endometrium slightly increased in patients with severe AS when they were injected with CD34+ or cultured autologous bone marrow stromal cells (128). In 2016, a case study reported the effect of injection of autologous peripheral blood CD133+ cells into the uterine spiral artery on endometrial reconstruction in 16 patients with refractory infertility (129). In the same year, 7 patients with severe IUA underwent autologous menstrual blood stromal cell transplantation, with two cases later successfully conceiving. In previous studies, transplantation of bone marrow/collagen complex into patients with severe IUA could help to achieve pregnancy and live birth after treatment (130). Our research group (131) recently proved that the combination of autologous adipose-derived mesenchymal stem cells (ADSCs) and gel can increase the endometrial thickness and reduce the fibrosis area through the BMP7-Smad5 pathway, resulting in successful conception.

Although transplantation with autologous MSCs is safer and more ethical than allogeneic MSCs, there are still some problems in the clinical applications. First, the number of extracted autologous MSCs is very limited as they can only be extracted from the host body (132). Second, after autologous MSCs are extracted, the *in vitro* culture cycle is long, which may not fully meet the needs of the body. Finally, there is a significant difference between the secretion and immune regulation of autologous MSCs (133).

## Conclusion and Prospective

Immune rejection is very common in allogeneic transplantation and is one of the top five causes of death in patients undergoing allogeneic transplantation (134). To avoid adverse reactions, MSCs with low immunogenicity have become a research hotspot in recent years. However, some scholars have proposed that allogeneic mesenchymal stem cells can still trigger immune rejection. At present, the mechanism by which autologous MSCs avoid immune rejection is unclear. However, the experimental results showed that when autologous MSCs and allogeneic MSCs were injected into the body for treatment, autologous MSCs survived longer and there were clusters of immune cells around allogeneic MSCs (113).

In addition, IUA is an important factor affecting female infertility. The regenerative ability of MSCs can be harnessed repair and restore the function of the fibrotic endometrium. However, from an ethical point of view, it is still unclear whether allogeneic MSCs affect the development of offspring after implantation in the human body. Therefore, autologous MSCs are recommended for the treatment of IUA. At present, there is no effective method to solve the problem of extracting fewer stem cells from autologous MSCs, but the increase in exosomes may not only solve the problem of immune rejection of allogeneic MSCs, but also that of the low number of autologous MSCs.

Further studies to overcome the immune rejection caused by allogeneic MSCs during the treatment process are necessary. Owing to the many sources of allogeneic MSCs and the high efficiency of *in vitro* culture, the treatment of immune rejection caused by allogeneic MSCs is still receiving widespread attention (135). On the other hand, an obvious solution is to immediately use autologous MSCs as a ready-made product. In addition, new products such as acellular exosomes and MSCs derived from human pluripotent stem cells (hPSCs) are exciting developments that are attracting significant attention (136).

## Author Contributions

J-mC, Q-yH, SL, and Q-yS contributed to the conception and design of the review. J-mC drafted and finalized the manuscript. SL and Q-yS contributed equally to writing the review. SL and Q-yS revised the manuscript and provided critical advice on the content of the manuscript. All authors contributed to the article and approved the submitted version.

## Funding

This work was supported by the Science and Technology Bureau of Quanzhou (grant number 2020CT003).

## Conflict of Interest

The authors declare that the research was conducted in the absence of any commercial or financial relationships that could be construed as a potential conflict of interest.

## Publisher’s Note

All claims expressed in this article are solely those of the authors and do not necessarily represent those of their affiliated organizations, or those of the publisher, the editors and the reviewers. Any product that may be evaluated in this article, or claim that may be made by its manufacturer, is not guaranteed or endorsed by the publisher.

## Glossary

**Table:**

ADSCs	Adipose-Derived Mesenchymal Stem Cells
APC	Antigen Presenting Cell
ATG	Antithymocyte Globulin
bFGF	Basic Fibroblast Growth Factor
BMP7	Bone Morphogenetic Protein-7
BMSCs	Bone marrow mesenchymal stem cells
CD	Cluster Of Differentiation
CSF-1	Colony Stimulating Factor-1
CTL	Cytotoxic Lymphocyte
CXCL12	C-X-C Motif Chemokine 12
CXCR4	C-X-C-motif chemokine receptor 4
DC	Dendritic Cells
EDSCs	Endometrialderived Stem Cells
EMT	Epithelial-to-Mesenchymal Transition
ERα	Estrogen Receptor α
FasL	Fas ligand
G-CSF	Granulocyte-Colony Stimulating Factor
GM-CSF	Granulocyte-Macrophage Colony Stimulating Factor
GVHD	Graft-Versus-Host Disease
GVHR	Graft-Versus-Host Response
HAECs	Human Amniotic Epithelial Cells
HEDSC	Human Endometrialderived Stem Cells
HGF	Hepatocyte growth factor
HLA	Human Leukocyte Antigen
HMSCs	Human Mesenchymal Stem Cells
hPSC	human pluripotent stem cells
IDO	Indoleamine 2,3-dioxygenase
IFN-γ	Interferon-γ
IGF-1	Insulin-like Growth Factor-1
IL-1	Interleukin-1
IL-10	Interleukin-10
IL-12	Interleukin-12
IL-17	Interleukin-17
IL-18	Interleukin-18
IL-1RA	Interleukin-1 Receptor Antagonist
IL-2	Interleukin-2
IL-4	Interleukin-4
IL-6	Interleukin-6
IL-25	Interleukin-25
IL-33	Interleukin-33
IUA	Intrauterine Adhesion
LIF	Leukemia Inhibitory Factor
LPS	Lipopolysaccharide
M2	Alternatively Activated Macrophages
MCP-1	Macrophage Chemoattractant Protein-1
M-CSF	Macrophage Colony Stimulating Factor
MHC	Major Histocompatibility Complex
MSCs	Mesenchymal Stem Cells
MMP	matrix metalloproteinase
NK cells	Natural Killer cells
OKT3	Anti-CD3 Antibody
OX40L	Oxford 40 ligand
PDGFR-β	Platelet-derived growth factor receptor-beta
PD-L1	Programmed Cell Death Ligand-1
PGE2	Prostaglandin E2
PIGF	Placental Growth Factor
SCF	Stem Cell Factor
SDF-1	Stromal Cell Derived Factor-1
Smad	Small Mothers Against Decapentaplegic
TCRA	Transcervical Resection Of Adhesions
Terg	Regulatory cells
TGF-β	Transforming Growth Factor-beta
TH1	T helper cell 1
TH17	T helper cell 17
TSLP	Thymic Stromal Lymphopoietin
TLR	Toll-like receptors
TNF-α	Tumor Necrosis Factor-α
TSC	Totipotent Stem Cell
TSG-6	Tumor Necrosis Factor a Stimulated Gene-6
UC-MSCs	Umbilical Cord-derived Mesenchymal Stem Cells
VEGF	Vascular Endothelial Growth Factor
